# *Crithmum maritimum* L.—Study on the Histochemical Localization of Essential Oil

**DOI:** 10.3390/plants13040550

**Published:** 2024-02-17

**Authors:** Velina Dzhoglova, Kalin Ivanov, Niko Benbassat, Yoana Georgieva-Dimova, Rayna Ardasheva, Diana Karcheva-Bahchevanska, Stanislava Ivanova

**Affiliations:** 1 Department of Pharmacognosy and Pharmaceutical Chemistry, Faculty of Pharmacy, Medical University of Plovdiv, 4002 Plovdiv, Bulgaria; 2 Department of Medical Physics and Biophysics, Faculty of Pharmacy, Medical University of Plovdiv, 4002 Plovdiv, Bulgaria; 3 Research Institute, Medical University of Plovdiv, 4002 Plovdiv, Bulgaria

**Keywords:** Apiaceae, *Crithmum maritimum* L., secretory ducts, essential oil, GC-MS, traditional medicine, histochemical analysis

## Abstract

*Crithmum maritimum* L. is a perennial halophyte plant that is a medicinal herb known by people from different cultures since ancient times. However, the therapeutic potential of this halophyte has not been completely investigated, and the scientific data on it are limited. The purpose of the present study was to estimate the chemical composition of the essential oil (EO) obtained from the aerial parts of *Crithmum maritimum* L. growing wild in Bulgaria, as well as the histolocalization of secretory structures for the synthesis and accumulation of volatile oils. The results obtained on the EO composition of Bulgarian *Crithmum maritimum* L. provide an opportunity to reveal potential future applications in various fields, such as medicine, pharmacy, agriculture, food, and the cosmetic industry. Gas chromatography with mass spectrometry was performed to assess the chemical profile of the isolated EO. The phenylpropanoid dillapiole was identified as the major compound in the EO, accounting for 34.09% of the total EO. Monoterpene hydrocarbons represented 62.07% of the total oil composition. *γ*-Terpinene, D-limonene, and *β*-pinene were the most abundant monoterpene hydrocarbons in the composition of the EO. In addition, histochemical localization of EO in the stem and leaves of *Crithmum maritimum* L. was carried out. The secretory structures were located in the cortical region of the stem and in the mesophyll tissues of the leaves in the form of secretory ducts. The performed histochemical analysis confirmed the lipophilic nature of the secretion from the duct cells. This is the first report related to the histolocalization and chemical composition of the EO from Bulgarian *Crithmum maritimum* L. Furthermore, our data indicate some potential possibilities for the evaluation of the therapeutic activity of the EO obtained from this plant species and outline its future applications as a therapeutic agent. Also, the EO from the studied halophyte plant has prominent potential to be used as a biopesticide, which is an environmentally friendly option compared to standard pesticides.

## 1. Introduction

**Figure 1:**
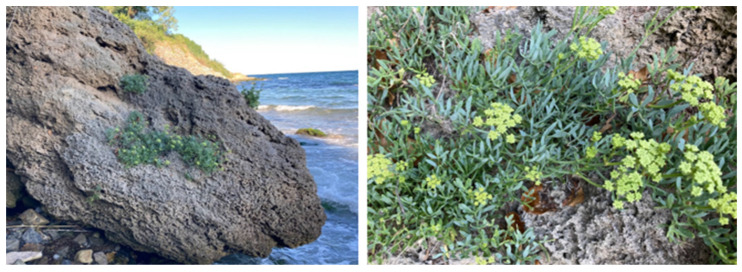
Wild-growing populations of *Crithmum maritimum* L. on coastal rocks in Sozopol, Bulgaria.

*Crithmum maritimum* L. is an aromatic halophyte plant that belongs to the botanical family Apiaceae (Umbelliferae) [1]. This halophyte is the only representative of the genus *Crithmum* [2]. *Crithmum maritimum* L. grows along the coasts of the Mediterranean Sea and the Black Sea [1,3]. The halophyte plant is also distributed along the Atlantic and Pacific coasts [3]. The plant is relatively known as Sea fennel, Rock samphire, and Crest marine because it grows on coastal rocks and sandy beaches (Figure 1) [4].

Halophyte plants, including *C. maritimum* L., have developed different mechanisms and produce specific phytochemicals to resist harsh conditions, which are the main characteristic of their natural habitats [5,6,7]. Halophyte plants are associated with high salt tolerance, and they could grow under other stressful conditions, such as drought, flooding, temperature fluctuations, and a high concentration of heavy metals [8,9,10].

*C. maritimum* L. is a perennial, rhizomatous plant up to 60 cm in height. Its stem is woody at the base and branched at the top. The leaves are succulent, pinnate, and glabrous, with thin cartilaginous margins, and the flowers are yellow–green [1,11]. The blooming period of this plant in Bulgaria begins in June and lasts until the end of August [12]. 

The taste of the plant is similar to that of typical salad crops, which makes the plant widely used in the cuisine of different countries [13]. The leaves of *C. maritimum* L. have nutritional value, and the halophyte plant is popular as food in Western Europe, especially in Portugal and France [14]. The aerial parts of the plant are included as a salad ingredient in the Mediterranean diet in countries such as Spain, Greece, and Tunisia [13,15]. In Turkish cuisine, the leaves and branches are used as a spice and an appetizer, and they are also included in the composition of traditional vinegar [16,17]. 

In folk medicine, people have used *C. maritimum* L. for its diuretic and carminative properties, and for scurvy treatment [18,19]. In the past, because of its vitamin C content, the extract from the aerial parts of the plant was used not only for the treatment, but also for the prevention of scurvy [19]. Sea fennel has also been widely known since ancient times for its anthelmintic properties [20]. Recent studies have shown that different extracts derived from the aerial parts exhibit antioxidant, anti-inflammatory, antiviral, antibacterial, antifungal, and cytotoxic activities [21,22,23,24]. The therapeutic properties of *C. maritimum* L. extracts are due to the rich phytochemical composition, which is presented mainly by phenolic acids and flavonoids [25]. It is also considered that *C. maritimum* L. extract could be used as an alternative to synthetic preservatives in foods and cosmetics [26]. As an aromatic plant, *C. maritimum* L. is a source of an EO composed of various biologically active substances [27]. The main compounds found in the EO are *γ*-terpinene, *α*-pinene, limonene, *p*-cymene, *β*-phelandrene, sabinene, and thymol methyl ether [27,28,29]. Studies from different countries related to the composition and biological activities of Sea fennel essential oil have proven its antioxidant, anti-inflammatory, and anticancer effects [27,30,31]. Sea fennel essential oil has also been found to have antibacterial and antifungal effects [3,32]. Data in the literature suggest that the EO from *C. maritimum* L. has significant antifungal activity against *Candida albicans*, *Cryptococcus neoformans*, and *Trychophyton rubrum* [27]. The essential oil also possesses antimicrobial activity, mainly against *Bacillus cereus* and *Pseudomonas aeruginosa* [3]. 

Many species of the plant family Apiaceae produce volatile oils in specialized internal secretory structures, mostly termed as canals or ducts, located in vegetative organs and in fruits [33]. There is no information on such secretory structures in the leaves and stem of *C. maritimum* L., except for one paper that pointed out the presence of secretory ducts in the leaves, discerning them from the crystalline structures formed by the flavonoids hesperidin and diosmin [34]. Furthermore, considering the variations in the composition of the EO in different geographical areas, during the data collection, no information was found regarding the phytochemical composition of the EO from this halophyte in Bulgaria.

Therefore, the purpose of the present study was to estimate the chemical composition of the EO from the aerial parts of the wild-growing *C. maritimum* L. in Bulgaria and to provide information on the secretory structures located in the leaves and stem of this species.

## 2. Results and Discussion

### 2.1. Microscopic Histochemical Analysis

**Figure 2:**
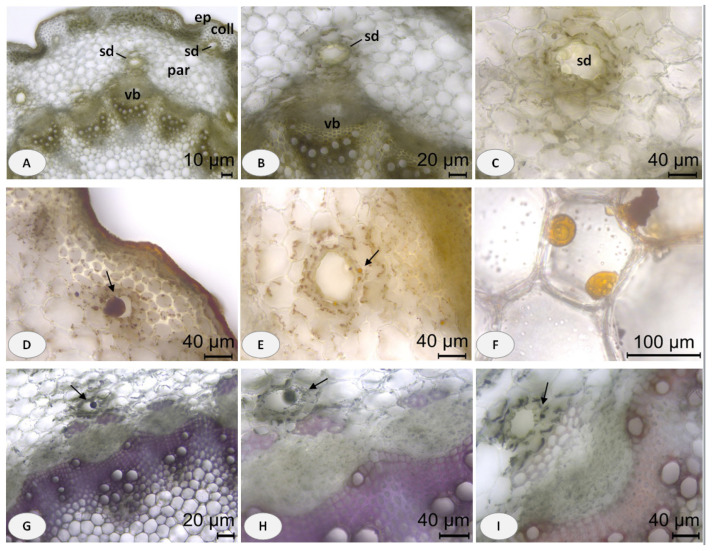
Stem transverse sections of *Crithmum maritimum* L.: (**A**) General aspect of unstained stem section showing the presence of secretory ducts (sd) embedded in collenchyma (coll) tissue under the epidermis (ep) and secretory ducts (sd) occurring in a ring in the cortical parenchyma (par) opposite the vascular bundles (vb); (**B**,**C**) details of stem secretory ducts (sd) filled with yellowish colored droplets; (**D**–**F**) positive orange—red staining of the lipid deposits and epithelial cells around the stem ducts; (**G**–**I**) positive blue staining of the surrounding epithelium and droplets in the stem secretory ducts; scale bars: (**A**) = 10 μm; (**B**,**G**) = 20 μm; (**C**–**E**,**H**,**I**) = 40 μm; (**F**) = 100 μm.

**Figure 3:**
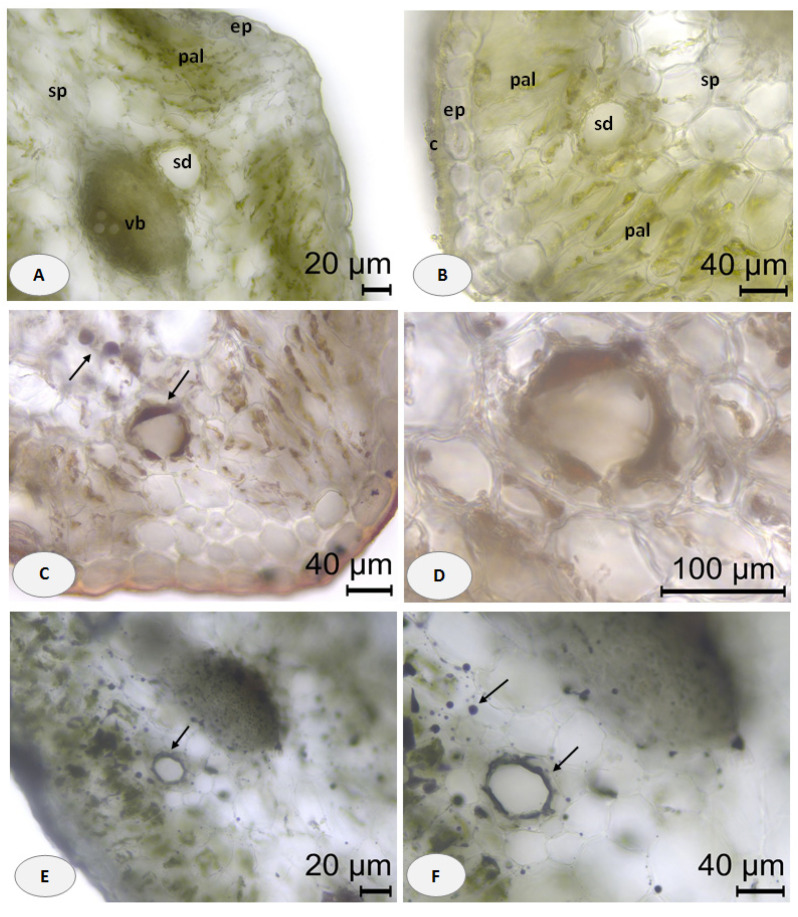
Leaf transverse sections of *Crithmum maritimum* L.: (**A**,**B**) general aspect of unstained leaf section showing epidermis (ep) covered with cuticle (c), palisade parenchyma (pal), and spongy parenchyma (sp) with secretory ducts (sd), accompanying the vascular bundles (vb); (**C**,**D**) details of leaf secretory ducts with positive orange–red staining of the lipid deposits; (**E**,**F**) details of leaf secretory ducts with positive blue staining droplets and surrounding epithelium; scale bars: (**A**,**E**) = 20 μm; (**B**,**C**) and (**F**) = 40 μm; (**D**) = 100 μm.

The microscopic observations of the examined *C. maritimum* L. organs revealed the presence of internal secretory structures identified as secretory ducts (Figure 2 and Figure 3). Such secretory ducts commonly occur in many species of the plant family Apiaceae and are a typical example of schizogenous cavities [35]. A diagnostic character of the stem’s transverse section was the presence of secretory ducts arranged in a concentric ring in the parenchyma of the cortex, often opposite the collateral vascular bundles. Secretory ducts were also observed exterior to the cortical parenchyma, embedded in the collenchyma tissue under the epidermis (Figure 2A). The yellowish oil droplets contained in the ducts were clearly visible in unstained stem sections mounted in water (Figure 2B,C). The oil droplets and related surrounding epithelial cells were considered to be lipophilic, as indicated by the orange–red staining with Sudan III (Figure 2D–F). The NADI reaction resulted in an intense blue staining of the secreted droplets, indicating the terpene content (Figure 2G–I).

In the transverse section of the leaf blade, an epidermis covered with a thin layer of cuticle was observed. The mesophyll comprised two or three layers of palisade parenchyma and three to five layers of dense spongy parenchyma. Secretory ducts were found in the mesophyll (Figure 3A,B); single and much larger ones were visible near the vascular bundles on the phloem side (Figure 3A). The application of Sudan III confirmed the lipid nature of the duct content as well as its presence in the surrounding epithelial cells (Figure 3C,D). The blue staining of secreted deposits through a NADI reaction indicated the presence of terpenes (Figure 3E,F).

In all the histochemical tests applied, a positive reaction was observed for the presence of lipids and terpenes in the leaves and stem of *C. maritimum* L., indicating the essential oil content.

### 2.2. Volatile Constituents of the Crithmum maritimum L. Essential Oil

**Figure 4:**
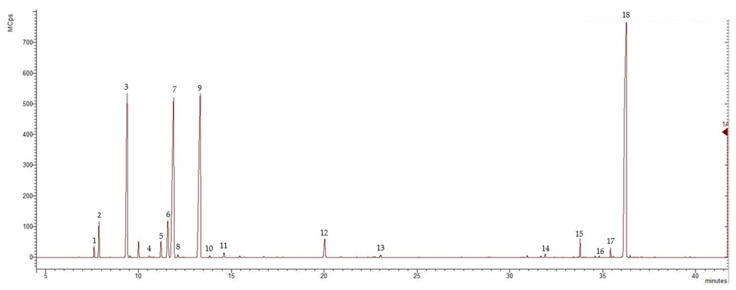
GC-MS chromatogram of the *Crithmum maritimum* L., where GCps—Giga Counts per second, and the numbers refer to the following compounds: 1-*α*-thujene, 2-*α*-pinene, 3-*β*-pinene, 4-octanal, 5-*α*-terpinene, 6-*p*-cymene, 7-D-limonene, 8-*trans*-*β*-ocimene, 9-*γ*-terpinene, 10-*trans*-sabinene hydrate, 11-*α*-terpinolene, 12-terpinene-4-ol, 13-thymol methyl ether, 14-*β*-caryophyllene, 15-elixene, 16-elemicine, 17-spathulenol, and 18-dillapiol.

The essential oil isolated through hydrodistillation from the aerial parts of *C. maritimum* L. was pale yellow in color, with a yield of 1.2%. The results from the analysis of the essential oil through GC-MS revealed the presence of 18 components, which represented 99.70% of the total oil content. The chemical composition of the EO is summarized in Table 1, and the chromatogram is presented in Figure 4.

It was established that the determined compounds belonged to different monoterpene classes, such as monoterpene hydrocarbons, oxygenated monoterpenes, and sesquiterpenes. There were also two substances which were derivates of phenylpropanoids and one substance belonging to the aldehyde class. The dominant compound detected in the EO was phenylpropanoid dillapiol, representing 34.09% of the total EO. The percentage composition of monoterpene hydrocarbons was 62.07%, and the major components were *γ*-terpinene, D-limonene, and *β*-pinene, respectively, representing 20.26%, 18.95%, and 16.04%. The oxygenated monoterpenes identified in the EO represented 2.03%, with terpinene-4-ol and thymol methyl ether constituents. 

**Figure 5:**
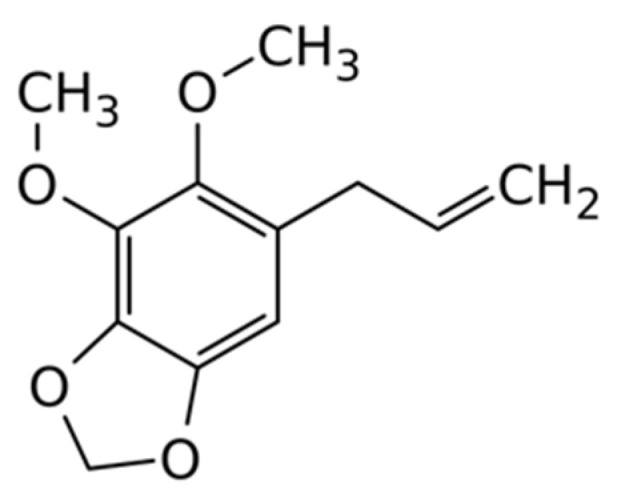
Structure of dillapiol.

Phenylpropanoid dillapiol (Figure 5) is the most abundant compound in the EO from *C. maritimum* L. collected from the Blak Sea region of Bulgaria. This substance could also be found in the EO obtained from different plants, such as *Piper aduncum*, *Apium nodiflorum*, *Anethum sowa*, and *Anethum graveolens* [36,37,38]. Recent studies have found that dillapiol could be associated with insecticidal activity; it was active against mosquito larvae and Colorado potato beetle in combination with the insecticide pyrethrum, which also has a plant origin [39,40]. In addition, it was revealed that dillapiole could increase the efficacy of not only plant-based pesticides, but also of synthetic ones, characterized by a high level of resistance [36,41]. The mechanism of action of dillapiole includes the inhibition of insect enzymes, with a role to eliminate their toxic metabolites [36]. This compound is of great interest because of the absence of toxic manifestations affecting the environment and human organisms [42]. It is established that dillapiol possesses protective effects against ethanol-induced gastric ulcer in animals [43]. 

Dillapiol and its semi-synthetic analogues are also associated with anti-inflammatory activity that is comparable to the anti-inflammatory effect of indomethacin, with the main difference being that dillapiol is a much safer alternative to conventional NSAIDs [43,44]. The antifungal activity of dillapiol has also been investigated, and it has been reported that dillapiol is characterized by antifungal properties against pathogenic fungal strains [32]. Other studies have reported that dillapiol exerts cytotoxic activity against a wide range of tumor cells by stimulating ROS production, leading to cell apoptosis [45,46,47]. Scholars have suggested that dillapiol has antiparasitic activity against parasites of the genus *Leishmania* [48]. Moreover, dillapiol increases the therapeautic effect of the antimalarial drug gedunin [49,50]. The efficacy of this combination is a result of the inhibition of the metabolism of poorly absorbable gedunin by dillapiol, leading to higher concentrations of gedunin in the blood [50].

The monocyclic monoterpene *γ*-terpinene is the second predominant substance in the EO from Bulgarian wild populations of *C. maritimum* L. The substance is present in the EO of various aromatic plant, including species of the botanical families Lamiaceae, Zingiberaceae, and Apiaceae [51,52,53,54]. Several studies have shown that, like other monoterpens, *γ*-terpinene exhibits anti-inflammatory activity in mice models of carrageenan-induced peritonitis by reducing edema, cells’ migration into damaged tissues, and production of proinflammatory cytokines [55,56,57,58]. *γ*-Terpinene also demonstrates analgesic activity when it is used for the treatment of neuropathic pain in animals, suggesting that it acts as a cannabinoid receptor agonist [59]. Additionally, previous studies have reported that γ-terpinene has antioxidant, anticancer, antibacterial, and antifungal properties [60,61,62,63,64,65,66]. 

D-limonene is an example of a well-studied substance of plant origin with multiple biological effects [67]. Essential oils from several citrus plants are rich in this monocyclic monoterpene; example are lemon, orange, grapefruit, lime, and grape [67]. D-limonene is widely used in the manufacture of cosmetic and food products, owing to its citrus aroma [68,69]. D-limonene is also used as a repellent and pesticide [70,71]. Potential therapeutic applications of D-limonene are based on studies demonstrating its antioxidant, anti-inflammatory, antibacterial, and antifungal properties [72,73,74,75,76]. Studies concerning the anticancer activity of D-limonene are particularly relevant, and the studies conducted have demonstrated the existence of therapeutic effect in various types of cancer, for instance, lung cancer, breast cancer, gastric cancer, and lymphoma [77,78,79,80,81]. *β*-Pinene is also found in relatively large quantities in the EO of *C. maritimum* L., growing on the Black Sea coast of Bulgaria. This compound exerts antimicrobial activity, which is expressed by suppressing the growth of *Staphylococcus aureus* and *Candida albicans* [82]. Moreover, *β*-pinene has anticancer, antioxidant, anti-inflammatory, gastroprotective, neuroprotective, and analgesic properties [83].

Further in vitro and in vivo studies in the future could be valuable in order to provide a vigorous understanding of the potential therapeutic applications of EO obtained from Bulgarian *C. maritimum* L.

### 2.3. Comparison of the Main Volatile Constituents of the EO of C. maritimum L. from Different Geographical Regions

Several studies have investigated the chemical composition and potential uses of *C. maritimum* L.’s EO. Data about the chemical composition of *C. maritimum* L.’s EO isolated from plants from different geographical regions are presented in Table 2. Although the harvesting regions varied, the data collected indicated the presence of many identical compounds, such as dillapiol, *γ*-terpinene, *β*-pinene, and *p*-cymene. However, the main compounds and their concentrations differ significantly. Compared to the EO isolated from plants in Cyprus, Turkey, Portugal, and Italy, the Bulgarian EO is the richest in dillapiol (34.09%). The EO obtained from cultivated *C. maritimum* L. in Tunisia was also rich in dillapiol [84]. In this study, the researchers reported that the EO isolated from the leaves of the plant contained the highest concentrations of dillapiol (41.35%). The stems were also found to be a source of the EO rich in dillapiol (31%), while the EO obtained from the flowers contained dillapiol at significantly lower levels (2.39%) [84]. The EO obtained from the wild-growing *C. maritimum* L. from Greece was also rich in dillapiol [85]. This study showed a comparison between the EO obtained from four populations of *C. maritimum* L. collected from different parts of the Amorgos Island in Greece [85]. The results of this investigation demonstrated that dillapiole was the compound present in the largest amount [85]. In contrast to *C. maritimum* L.’s EO from Bulgaria, Tunisia, Italy, Portugal, Turkey, Cyprus, and Greece, the EO from *C. maritimum* L. obtained from Croatia demonstrated the absence of dillapiol [86]. The major substance in this EO was sabinene (42.55–51.47%) and D-limonene (36.28–43.58%) [86].

Compared to the essential oils from other geographical regions, the Bulgarian EO is poor in the natural bicyclic monoterpene sabinene. Unlike other countries, the results show that the Bulgarian EO has small quantities of the oxygenated monoterpene thymol methyl ether and the monoterpene hydrocarbon *p*-cymene. 

Compounds isolated from essential oils have huge potential for application, not only in the pharmaceutical and food industries, but also in the agricultural and cosmetics industry. These compounds could be included in the formulation of biopesticides, nutritional supplements, and cosmetic and pharmaceutical products, providing beneficial therapeutic effects and low toxicity. Natural compounds could also be effective synergists when combined with other synthetic medicines or phytochemicals, enhancing their biological activity [91,92,93,94,95,96].

Strategies for the cultivation of Bulgarian *C. maritimum* L. could be especially beneficial for the production of EOs with specific characteristics and biologically active compounds.

Currently, one of the main challenges in the utilization of halophyte plants is the crop breeding. However, for a better exploration of their potential, cultivars with predictable phytochemical compositions need to be established. Some genomics-assisted breeding strategies could make a great contribution to enhancing the efficiency of crop breeding and the creation of better varieties [97].

## 3. Materials and Methods

### 3.1. Plant Material

The aerial parts of flowering *C. maritimum* L. were collected in July 2023 in the city of Sozopol, “Kavatsite” area, Bulgaria (42°23′50.4″ N 27°42′25.8″ E). A voucher specimen of the species (No: 063393) was deposited in the Herbarium of the Agricultural University—Plovdiv (SOA).

### 3.2. Chemicals and Reagents

To determine the retention indices (RI), the following hydrocarbons were used: nonane (≥99%), decane (≥99%), undecane (≥99%), dodecane (99%), tridecane (≥99%), tetradecane (≥99%), and hexadecane (≥99%), purchased from Merck KGaA (Darmstadt, Germany). Dichloromethane (Sigma Aldrich, Steinheim, Germany) was used for the dilution of the EO.

### 3.3. Microscopic Histochemical Analysis

For general histological observations, the plant material was fixed in a solution of 60% ethanol and glycerol in a ratio of 9:1 as a softening procedure of the tissues prior to sectioning [98]. Temporary preparations of hand-cut transverse sections were made from the middle part of the leaf and stem of the plant. For the presence of lipids in tissues, the sections were treated with a Sudan staining solution (Sudan III in 70% ethanol) for 20 min, rinsed in 50% ethanol, and mounted in 50% glycerol [99]. To reveal the presence of terpenes in the tissues, the sections were treated with a NADI staining solution (0.5 mL of 0.1% *α*-naphthol, 0.5 mL of 1% *N*,*N*-dimethyl-*p*-phenylenediamine, and 49 mL of 0.1 M sodium phosphate buffer, pH 7.2) for 1 h, rinsed in 0.1 M sodium phosphate buffer, pH 7.2 for 2 min, and immediately analyzed under a microscope [100]. The image analysis was performed using a light microscope (Leica DM 2000 LED, Leica Microsystems, Wetzlar, Germany) equipped with a digital camera (Leica DMC 2900) and software for processing images (Leica Application Suite, LAS).

### 3.4. Isolation of the Essential Oil

A quantity of 100 g of dried *Crithmum maritimum* L., aerial parts, was subjected to hydrodistillation for 4 h to 100 °C using a Clevenger-type apparatus to extract the essential oil. The collected oil was dried over anhydrous sodium sulfate to remove residual water droplets and then stored in dark glass vials at 4 °C until GC-MS analysis. The essential oil hydrodistillation yield (%) was measured based on the dry weight of the plant.

### 3.5. Chromatographic Conditions

The analysis of the EO was performed using gas chromatography with mass spectrometry (GC-MS). For the GC-MS analysis, we used a Bruker Scion 436-GC SQ MS (Bremen, Germany) equipped with a ZB-5MSplus fused silica capillary column (0.25 m film thickness and 30 m 0.25 mm i.d.). Helium was used as a carrier gas with a constant flow rate of 1 mL/min. The injection volume was 1 µL. The split ratio of the injector was 1:20. The oven temperature was set at 50 °C for 1 min, increased to 70 °C at a rate of 4 °C/min, increased to 110 °C at a rate of 2 °C/min, held for 1 min, and then increased to 220 °C at a rate of 8 °C/min for 1 min. The temperature of the injector was 250 °C, and the detector temperature was set to 300 °C. The mass spectra were in a full-scan mode with a mass range of 50–350 *m*/*z*. The identification of the separated components of the essential oil was achieved by comparing their MS spectra and retention indices (RI) with spectral data within the Wiley NIST11 Mass Spectral Library (NIST11/2011/EPA/NIH) and data in the literature. The RI values were calculated from the retention times of the C8-C30 *n*-alkane series injected under the same conditions described above. The experiment was performed in triplicate. Standard deviations (SDs) did not exceed 2% of the obtained values of each component.

## 4. Conclusions

In the present paper, a detailed analysis of the histolocalization of secretory structures in the stem and leaves of *C. maritimum* L. is described for the first time and supported by photomicrographs. In addition, no data on the chemical profile of the essential oil of *C. maritimum* L. growing wild in Bulgaria have been previously provided. The performed GC-MS analysis revealed the presence of volatile compounds, such as monoterpene hydrocarbons, oxygenated monoterpenes, phenylpropanoids, and sesquiterpenes. Eighteen substances were found to be present in the EO, accounting for 99.70% of the total oil. Representing 34.09%, dillapiol was the dominant compound in the EO. Our study also showed the presence of *γ*-terpinene, D-limonene, and *β*-pinene as other major compounds. Due to the presence of bioactive components in the EO’s composition, *C. maritimum* L. has the potential to be included in the formulation of novel pharmaceutical products. Also, this EO could be a beneficial source of dietary supplements and cosmetic products. The EO of this halophyte plant species has the potential to be used in a variety of therapeutic areas including symptoms and conditions, such as inflammation, bacterial and fungal infections, oxidative stress, and neoplastic diseases. Furthermore, the EO can provide an eco-friendly alternative to synthetic pesticides as a biopesticide without harmful effects related to the environment and humans.

## Figures and Tables

**Table 1:** Volatile constituents of the EO of *Crithmum maritimum* L. from Bulgaria as a percentage of total EO.

No	Compound	Retention Time (min)	RI	Kovats RI	Formula	Class of Compound	% of Total EO
1	*α*-thujene	7.615	929	924	C10H16	MH	0.54
2	*α*-pinene	7.876	934	932	C10H16	MH	1.76
3	*β*-pinene	9.393	972	974	C10H16	MH	16.04
4	octanal	10.59	990	998	C8H16O	SFA	0.11
5	*α*-terpinene	11.212	1018	1014	C10H16	MH	1.02
6	*p*-cymene	11.581	1020	1020	C10H14	MH	2.97
7	D-limonene	11.898	1029	1024	C10H16	MH	18.95
8	*trans*-*β*-ocimene	12.126	1051	1044	C10H16	MH	0.18
9	*γ*-terpinene	13.312	1059	1054	C10H16	MH	20.26
10	*trans*-sabinene hydrate	13.836	1068	1065	C10H18O	MO	0.23
11	*α*-terpinolene	14.607	1089	1086	C10H16	MH	0.35
12	terpinene-4-ol	20.029	1176	1180	C10H18O	MO	1.59
13	thymol methyl ether	23.043	1237	1232	C11H16O	MO	0.21
14	*β*-caryophyllene	31.899	1416	1417	C15H24	S	0.17
15	elixene	33.777	1488	1490	C15H24	S	0.79
16	elemicine	34.803	1520	1530	C12H16O3	PP	0.06
17	spathulenol	35.417	1577	1577	C15H24O	S	0.38
18	dillapiol	36.259	1633	1620	C12H14O4	PP	34.09
	Terpene classes						
	MH-monoterpene hydrocarbons						62.07
	MO-oxygenated monoterpenes						2.03
	PP-phenylpropanoids						34.15
	S-sesquiterpenes						1.34
	Aldehyde class						
	SFA-saturated fatty aldehydes						0.11
	Total identified						99.70

The percentage of relative peak area is the average value of three measurements. The standard error of the mean was eliminated, and it was not more than 2%.

**Table 2:** Comparison of the main volatile constituents of the EO of *Crithmum maritimum* L. from different geographical regions.

Plant Collecting Region	Plant Materials	Collecting Period	Main Volatile Compounds	Other Volatile Compounds	Ref.
Bulgaria	Aerial parts	July	dillapiol (34.09%), *γ*-terpinene (20.26%), D-limonene (18.95%), *β*-pinene (16.04%)	*p*-cymene (2.97%)	Present study
Cyprus	Leaves	May	*γ*-terpinene (39.03%), *β*-phellandrene (22.06%)	carvacrol methyl ether (10.4%), (Z)-*β*-Ocimene (8.2%), *p*-cymene (6.4%)	[87]
Turkey	Aerial parts	August	sabinene (26.9%), D-limonene (24.2%), *γ*-terpinene (19.3%)	terpinen-4-ol (9.0%), methyl chavicol (3.4%)	[88]
Italy	Comparison between EO isolated from stems, leaves, flowers and seeds.	Different periods	*γ*-terpinene (41–68%), sabinene (1.4–30%), methyl thymolol (12–17.7%), 1,8-cineole (0.9–15.6%)	*p*-cymene (4.8–10%), dillapiol (0.5–9.5%)	[89]
Tunisia *	Comparison between EO isolated from stems, leaves, flowers and seeds.	February (EO isolated from stems and leaves), June (EO isolated from flowers and seeds).	dillapiole (2.39–41.35%), thymyl methyl ether (20.13–34.75%), *p*-cymene (4.83–22.08%), *γ*-terpinene (22.54–43.29%)	*β*-Pinene (1.08%)	[84]
Portugal	Comparison between EO isolated from inflorescence, leaves, stems.	Different periods (from July until November).	sabinene (8.4–40.1%), *γ*-terpinene (43.5–22.3%), thymol methyl ether (12.1–16.5%)	dillapiol (0.5–8.3%)	[90]
Greece	Comparison between EO isolated from inflorescence, leaves, stems from four populations.	August	dillapiole (0–48.8%), methyl thymol (4.4–41.0%), *γ*-terpinene (6.9–28.6%)	*p*-cyrnene (0.8–15.8%), terpinen-4-0l (3.9–16.0%), sabinene (1.1–24.7%), *β*-phellandrene (0–10.3%)	[85]
Croatia	Comparison between EO isolated from inflorescence, leaves, stems.	September	sabinene (42.55–51.47%), D-limonene (36.28–43.58%)	terpinen-4-ol (3.53–10.35%), *γ-*Terpinene (2.79–5.28%)	[86]

* The plants were cultivated.

## Data Availability

Data are contained within the article.
